# Physicians’ therapeutic practice and compliance of diabetic patients attending rural primary health care units in Alexandria

**DOI:** 10.4103/1319-1683.74325

**Published:** 2010

**Authors:** Nahla Khamis R. Ibrahim, Saeid G. Attia, Sunny A. Sallam, Ebtisam M. Fetohy, Fatihey El-Sewi

**Affiliations:** 1 *Department of Epidemiology, High Institute of Public Health, Alexandria University, Egypt*; 2 *King Abdul-Aziz University, Jeddah, Kingdom of Saudi Arabia, Egypt*; 3 *Alexandria Directorate of Health Affairs, High Institute of Public Health, Alexandria University, Egypt*; 4 *Department of Health Administration and Behavioral Sciences, High Institute of Public Health, Alexandria University, Egypt*; 5 *Faculty of Medicine, Alexandria University, Egypt*

**Keywords:** Compliance, diabetes, doctor-patient relationship, knowledge, skills

## Abstract

**Objectives:**

The objectives of the study were to investigate physician’s therapeutic practice and the compliance of diabetic patients attending rural primary health units in Alexandria.

**Material and Methods:**

A cross-sectional study was conducted and a multistage stratified random sample method was used for the selection of 600 diabetic patients. Data were collected by means of an interviewing questionnaire, an observation checklist, review of prescriptions and laboratory investigations. A scoring system was made for a diabetic patient’s knowledge and skills, patient’s compliance, doctor-patient relationship, and glycemic control.

**Results:**

About 57% always took their medication as prescribed by doctor and on time, only 2.2% always complied with dietary regimen while no one reported regular compliance with exercise regimen. Complications of the regimen was the commonest cause (63.3%) of noncompliance. A highly statistically significant difference was found between compliance with all regimens and patient’s knowledge of diabetes. The scores for doctor-patient relationship were all unsatisfactory. Results of glycosylated hemoglobin (HbA1c) revealed that metabolic control of four-fifth of the patients was satisfactory, 12% had fair and 8% had poor metabolic control.

**Conclusions:**

Patient’s compliance with most of the diabetes regimen was low. Doctor-patient relationship and patient’s compliance should be improved by conducting educational and training programs.

## INTRODUCTION

Diabetes is a major global public health problem, with challenging epidemiology.[1,2] This threat to global health is escalating and rapidly worsening.[3,4] The increasing prevalence of diabetes mellitus, the emergence of complications of diabetes as a cause of early morbidity and mortality, and the enormous mounting burden on health care systems make diabetes a priority health concern.[5,6] Between 1995 and 2025, the number of the adult population affected by diabetes mellitus in developing countries is projected to grow by 170%, from 84 to 228 million people.[6,7] The prevalence of diabetes in some Eastern Mediterranean countries is among the highest in the world. The cause of this high prevalence is the result of the many social and economic changes that have occurred in the majority Eastern Mediterranean nations in the last three decades.[4,5]

The care of diabetes involves some changes in lifestyle, including dietary habits and regular intake of medications.[8] Successful management of diabetes relies on patients’ self-care.[9] Compliance is a key element in health care and affects all of its areas.[10] The degree of patients compliance (adherence) to diabetes self-care is the extent to which patients carry out the set of daily activities recommended to them by a health care professional as a means of managing their diabetes. These include dietary style, exercise, taking medication, monitoring of blood glucose, foot care, as well as the timing and integration of all of these activities.[11]

The problem of poor compliance or adherence to prescribed treatments is very complex.[12] Affecting compliance are many factors relating to the patient, the disease, the physician, and the family.[8] Patients and caregivers interpret signs and signals differently. These differences in perspective are not inherently problematic. They frequently become so when patients do not meet the goals and expectations of their health care providers.[12] The development of more-effective behavioral strategies to promote adherence is needed to achieve maximum benefit to the patient. Measures that focus on patients’ perceptions can be effective in altering behavior.[9]

The physician-patient interview is the key component of all health care, particularly of primary medical care. A good provider-patient relationship is especially important in the management of chronic diseases such as diabetes. Existing research on doctor-patient relationship is limited.[13] This study is urgent because not much research has been done on diabetic therapeutic practice and compliance of diabetic patients. The objectives of the study were to investigate physician’s therapeutic practice in diabetes mellitus and determine the degree of compliance and causes of noncompliance of diabetic patients attending rural primary health care (PHC) units in Alexandria.

## MATERIALS AND METHODS

A cross-sectional study was conducted on diabetic patients attending rural PHC units in Alexandria during the period 1999 to 2004. Following the WHO recommendations for the investigation of drug use in health facilities, the sample of diabetic patients was taken. This states that studies describing current treatment practice should include at least 600 encounters from sampled health facilities comprising at least 30 encounters from each facility.[14] Accordingly, 20 rural PHC units were randomly selected from all PHC units in Alexandria. Multistage stratified random sample method was used. Stratification considered the type of district (desert or agricultural) of units. All diabetic patients attending the rural unit on the day of interview who agreed to participate in the study were included. There were ethical considerations of confidentiality and the freedom to participate or not.

### Data collection tools

The data were collected for each patient by completing an interviewing questionnaire, filling an observational checklist, and reviewing prescription forms and laboratory investigations. The questionnaire was validated.

#### Interviewing questionnaire

The questionnaire completed during an interview by the researcher with the patient covered the following data:

- Personal, socio-demographic data: data about age, gender, occupation, marital status, and education.
- Diabetes history: Duration of diabetes, course of disease (controlled, uncontrolled, or complicated) and type of regimen (insulin or oral hypoglycemic drugs).
- Compliance: Questions on compliance dealt with the following:
  - Medication: taking medication as prescribed and taking medication on time.
  - Dietary: adherence to dietary regimen
  - Exercise: Taking a 20-minute walk a day
  - Self care: eye, foot, dental, and skin care.
  - Causes of noncompliance (if any): the causes of noncompliance were economic, lack of knowledge of the importance of compliance and difficulty of the regimen, etc.
- Knowledge and skills concerning diabetes mellitus:
  - Knowledge: Patients were asked about symptoms of diabetes, predisposing factors, acute and chronic complications, management, and site of insulin injection.
  - Diabetic patients’ skills: Questions included patient’s skills for monitoring blood and urine glucose level, skills related to insulin use (rolling the vial, dose accuracy, and storage), foot care, and diet regulation.

#### Observation checklist

Health care providers and patients were observed by the researcher during the patient’s consultation with the doctor and the observational checklist was completed as follows:

- Examination of the patient: the investigator observed the doctor during the provision of medical care for the diabetic patient. Measurement of body weight, blood pressure, examination of the nervous system, feet and complications were observed and recorded in the checklist.
- Ordered laboratory investigations: Investigations for ordered blood and urine were checked.
- Health education provided for patient: This included education on dietary regimen, conduct of physical exercise, and self care (foot care, eye, skin and dental care and general hygiene). This was measured on a two-point scale (yes or no).

The number of prescribed drugs was recorded, and the consultation time (the time taken by the doctor with every patient attending rural health units including examination, education, and drug prescription) was calculated. The conduct of patient’s examination, health education on diabetes, instructions on drug use, and education on self care were also determined.

#### Prescription review

Prescription forms were reviewed to find out types of antidiabetic drugs prescribed (tablets or injections), their route of administration, and the prescription of other drugs.

#### Conduction of laboratory investigations

Laboratory investigation including urine test for presence of glucose, and blood test for the level of glycosylated hemoglobin (HbA1c) were done for the patients. This test was done for a subsample of 100 randomly selected patients. The cost of HbA1c was borne by the investigator.

### Data management

The data were checked, reviewed, and analyzed using SPSS version 10.

The following scores were calculated:

a. Patient’s knowledge score. This consisted of 14 questions. For each question, two points were given for giving ≥ half of the correct answer, one point for giving < half of the answer, and those who did not know or gave the wrong answer scored zero.

The total score for knowledge was 28 points. It was categorized into:

- Poor, <14
- Fair, 14 to 20 points.
- Satisfactory, 21 to 28 points

b. Compliance: Eight questions on compliance were asked. The eight questions were divided as follows: two questions on compliance with medication, one for dietary compliance, one for exercise compliance, and four for self-care compliance. Every question in the questionnaire was scored as follows:

- Never complies, 0 point.
- Sometimes complies, 1 point.
- Always complies, 2 points.

The total score of compliance was 16 points and classified into:

- Poor, <6 points
- Fair, 6 to 12 points
- Good, 13 to 16 points.

c. Doctor-patient relationship: Nineteen items were designed in the observational checklist to record the management of patients by PHC doctors. Every item was scored as yes (one point) or no (zero point). The total score for practice was 19 points and was categorized into:

- Poor, <6 points.
- Fair, 6 to 12 points
- Good, >13 points

d. Glycemic control:[15] The glycosylated hemoglobin (HbA1c) percentage was entered as a continuous variable, and further categorized into three levels as follows:

- Poor, >8%
- Fair, 7 to 8%
- Good, <7%

The mean and standard deviation were calculated from univariate analysis. Relationships between the degree of compliance (with drugs, diet, and exercise) and independent variables related to patients, disease, and care characteristics were determined using the Chi-squared and Fisher’s exact tests. Statistical significance was set at *P*≤0.05.

## RESULTS

The personal and socio-demographic characteristics of the sample of 600 diabetic patients indicated that men represented 48.3% of the sample while women formed 51.7%; the men to women ratio being 1 : 1.1. The age of diabetic patients ranged from 25 to 81 years, with a mean age 47.68 ± 11.94 years. Married patients comprised 83.0%, and 10.8% were single. About one-third (34.5%) of diabetic patients were illiterate and 41.2% were manual workers.

An analysis of results revealed that about two-third (64.3%) of the sample got their knowledge from physicians, 19.3% from nurses, 18.7% from relatives, and 4.8% from other diabetic patients.

Most patients gave more than one cause for non-compliance. About two-third of the patients (63.3%) said that the noncompliance was because they did not understand the drugs. Lack of knowledge about drugs was mentioned by 51.3%, whereas the reasons were financial for 27% of the patients.

Table 1 illustrates the relationship between different types of compliance with diabetes regimen and gender. No difference of statistical significance was found, giving all items of compliance and genders (*P*>0.05). It is also apparent from the table that 57.6 and 57.5% of all patients always took their medications as prescribed and on time, respectively. However, only 2.2% of patients always complied with dietary regimen, while none of the patients always complied with exercise regimen.

**Table 1:** Relationship between different types of compliance and gender of diabetic patients

Gender Compliance	Male		Female		Total		*P*
	No.	%	No.	%	No.	%	
Taking medication as prescribed							
Never	5	26.3	14	73.7	19	3.2	>0.05
Sometimes	116	49.4	119	50.6	235	39.2	
Always	169	48.8	177	51.2	346	57.6	
Taking medication on time							
Never	5	26.3	14	73.7	19	3.2	>0.05
Sometimes	118	50.0	118	50.0	236	39.3	
Always	167	48.4	178	51.6	345	57.5	
Dietary control							
Never	14	41.2	20	58.8	34	5.7	>0.05
Sometimes	273	49.4	280	50.6	553	92.1	
Always	3	23.1	10	76.9	13	2.2	
Exercise control							
Never	282	48.3	301	51.7	583	97.2	>0.05
Sometimes	8	47.1	9	52.9	17	2.8	
Total	290	48.3	310	51.7	600	100.0	

Table 2 indicates that the high percentage of patients who always took medications as prescribed (37.0%) and on time (37.7%) were 35 to <45 years old. Statistical significant differences were found between age of the patients and compliance with taking medication as prescribed (*P*<0.05), and with dietary regimen (*P*<0.01).

**Table 2:** Relationship between different types of compliance and age group of diabetic patients

Age Compliance	25-		35-		45-		55+		Total		*P*
	No.	%	No.	%	No.	%	No.	%	No.	%	
Taking medication as prescribed											
Never	5	26.3	2	10.5	7	36.8	5	26.3	19	3.2	0.016
Sometimes	35	14.9	126	54.6	41	17.4	33	14	235	39.2	
Always	46	13.3	128	37.0	98	28.3	74	21.4	346	57.6	
Taking medication on time											
Never	5	26.3	2	10.5	7	26.3	5	26.3	19	3.2	>0.05
Sometimes	28	11.9	124	52.5	45	19.1	39	16.5	236	39.3	
Always	53	15.4	130	37.7	94	27.2	68	19.2	345	57.5	
Dietary control											
Never	2	5.9	13	38.2	9	26.5	10	29.4	34	5.7	0.004
Sometimes	84	15.2	240	43.3	131	23.7	98	17.8	553	92.1	
Always	0	0	3	23.0	6	46.2	4	30.8	13	2.2	
Exercise control											
Never	85	14.6	248	42.5	142	24.4	108	18.5	583	97.2	>0.05
Sometimes	1	5.9	8	47.1	4	23.5	4	23.5	17	2.8	
Total	86	14.3	256	42.7	146	24.3	112	18.7	600	100	

Results of the study showed that there was no significant relationship between the various aspects of compliances and socio-demographic characteristics of the patients such as education, occupation, marital status, and income.

Table 3 reveals that the majority of patients who always complied with instructions on taking medication as prescribed (94.5%), taking it on time (93.7%) and all of those who always complied with dietary regimen had fair knowledge score about diabetes mellitus. The relation between compliance and diabetic patient’s knowledge was highly significant for all types of regimens.

**Table 3:** Relationship between types of compliance and patients’ diabetic knowledge

Compliance	Diabetic patients’ knowledge								*P*
	Poor		Fair		Satisfactory		Total		
	No.	%	No.	%	No.	%	No.	%	
Taking medication as prescribed									
Never	5	26	14	74	0	0	19	3.2	<0.000
Sometimes	5	2.1	223	94.9	7	3	235	39.2	
Always	2	0.6	327	94.5	17	4.9	346	57.6	
Taking medication on time									
Never	5	26.0	14	74.0	0	0.0	19	3.2	<0.000
Sometimes	2	0.9	229	97.0	5	2.1	236	39.3	
Always	5	1.4	321	93.7	19	4.9	345	57.5	
Dietary control									
Never	5	15.0	29	85.0	0	0	34	5.6	<0.000
Sometimes	7	1.3	522	94.3	24	4.3	553	92.2	
Always	0	0	13	100	0	0	13	2.2	
Exercise control									
Never	12	2.1	552	94.7	19	3.2	583	97.2	<0.000
Sometimes	0	0	12	70.6	5	29.4	17	2.8	
Total	12	2	564	94	24	4	600	100.0	

Table 4 illustrates the relationship between types of compliance and patient’s skills toward diabetes. It is apparent from the table that the majority of patients had poor skills, irrespective of type of compliance. There was statistical significant difference only between taking medication as prescribed (*P* = 0.007) and the patients’ diabetic skills.

**Table 4:** Relationship between different types of compliance and patients’ skills toward diabetes

Compliance	Patient’s skills								*P*
	Poor		Fair		Satisfactory		Total		
	No.	%	No.	%	No.	%	No.	%	
Taking medication as prescribed									
Never	19	100	0	0.0	0	0.0	19	3.2	0.007
Sometimes	210	89.3	19	8.1	6	2.6	235	39.2	
Always	325	94.0	15	4.3	6	1.7	346	57.6	
Taking medication on time									
Never	19	100.0	0	0.0	0	0.0	19	3.5	0.091
Sometimes	218	92.4	14	5.9	4	1.7	236	39.3	
Always	317	91.9	20	5.8	8	2.3	343	57.5	
Dietary control									
Never	32	94.1	2	5.9	0	0.0	34	5.7	>0.05
Sometimes	510	92.2	31	5.6	12	2.2	553	92.1	
Always	12	92.3	1	7.7	0	0.0	13	2.2	
Exercise control									
Never	543	93.2	31	5.3	9	1.5	583	97.2	>0.05
Sometimes	11	64.8	3	17.6	3	17.6	17	2.8	
Total	554	92.3	34	5.7	12	2.0	600	100.0	

On doctor-patient relationship, one-third of patients had had health education about dietary regimen while a minority had had education on exercise, dental, eye, and skin care, and on side effects of drugs and precautions to be taken. For most of the patients, consultation time was no more than five minutes.

Table 5 illustrates the relationship between compliance with different diabetes regimen and doctor- patient relationship. It is apparent from the table that none of the doctor-patient relationships had a satisfactory score. It was found that 93.4% of those who always took their medication as prescribed, 92.5% of those who always took medication on time, and 69.2% who always complied with dietary regimen had fair doctor-patient relationship. Statistical significant difference was found only between dietary control (*P* = 0.001) and the degree of doctor-patient relationship.

**Table 5:** Relationship between types of compliance and doctor-patient relationship

Compliance	Doctor-patient relationship						*P*
	Poor		Fair		Total		
	No	%	No	%	No	%	
Taking medication as prescribed							
Never	0	0.0	19	100.0	19	3.2	>0.05
Sometimes	14	6.0	221	94.0	235	39.2	
Always	23	6.6	323	93.4	346	57.6	
Taking medication on time							
Never	0	0.0	19	100.0	19	3.2	>0.05
Sometimes	11	4.7	225	95.3	236	39.3	
Always	26	7.5	319	92.5	345	57.5	
Dietary control							
Never	2	5.9	32	94.1	34	5.7	0.001
Sometimes	31	5.6	522	94.4	553	92.1	
Always	4	30.8	9	69.2	13	2.2	
Exercise control							
Never	35	6.0	548	94.0	583	97.2	>0.05
Sometimes	2	11.8	15	88.2	17	2.8	
Total	37	6.2	563	93.8	600	100.0	

Table 6 shows results of analysis of the level of glycosylated hemoglobin (HbA1c) for the subsample of diabetic patients (100 patients). It illustrates that metabolic control of four-fifth (80%) of the patients was satisfactory, 12% was fair, and 8% was poor. The majority of patients who always complied with taking medications as prescribed (84.5%), taking medications on time (83.0%), and all of those who always complied with dietary regimen (100%) obtained satisfactory glycosylated hemoglobin level (better glycemic control).

**Table 6:** Relationship between different types of compliance and score of glycosylated hemoglobin (HbA1c) among diabetic patients

Compliance	Glycosylated hemoglobin (HbA1c)								*P*
	Poor		Fair		Satisfactory		Total		
	No.	%	No.	%	No.	%	No.	%	
Taking medication as prescribed									
Never	0	0.0	0	0.0	1	100	1	1.0	>0.05
Sometimes	4	9.8	7	17.1	30	73.2	41	41.0	
Always	4	6.9	5	8.6	49	84.5	58	58.0	
Taking medication on time									
Never	0	0.0	0	0.0	1	100	1	1.0	>0.05
Sometimes	3	7.5	7	17.5	30	75.0	40	40.0	
Always	5	8.5	5	8.5	49	83.0	59	59.0	
Dietary control									
Never	0	0.0	1	33.3	2	66.7	3	3.0	>0.05
Sometimes	8	8.3	11	11.5	77	80.2	96	96.0	
Always	0	0.0	0	0.0	1	100	1	1.0	
Exercise control									
Never	8	8.2	12	12.4	77	79.4	97	97.0	>0.05
Sometimes	0	0.0	0	0.0	3	100	3	3.0	
Total	8	8.0	12	12.0	80	80.0	100	100	

## DISCUSSION

Compliance of diabetic patients with medical advice is essential for the control of the disease.[11] Although adherence to medication is one of the most important aspects of the management of diabetes mellitus, low rates of adherence have been documented.[16] Results of the present study revealed that about 57% of patients always took their medication as prescribed and on time. Results of the study by Kravitz *et al*.[17] in Scotland found that 91% of the diabetic patients reported that they actually took their medication as prescribed. This difference in results may be due to differences in awareness of importance of compliance among diabetic patients in the two countries.

The results on the dietary compliance of the current study illustrated that 92.1 and 2.2% sometimes and always, respectively, complied with the dietary regimen. Khattab *et al*. found that there was good compliance by 40% of Saudi patients with dietary regimen.[8] However, results of the present study revealed that the lowest compliance was for the exercise regimen, which agrees with results of study by Kamel *et al*.[18]

On the causes of noncompliance, the present study revealed that the reason for noncompliance by 27% of the patients was financial. A study conducted in Louisiana, USA, found that the difficulty with noncompliance as reported by more than 75% of the sample included the costs of the medication.[11] The cause of this discrepancy between the two studies may be because the Louisiana study was conducted among low-income individuals or because subjects in the current study receive their medications free of charge from the PHC.

Results of the present study showed that there was minimal gender difference with no statistical differences in adherence to different aspects of the diabetic regimen. This is similar to the results of a study reported from some European clinics that found minimal gender difference in adherence to the component of self-care.[19]

Results of the current study showed that there is no significant relationship between the various aspects of compliance and the socio-demographic characteristics of the patients such as education, occupation, and marital status. This is comparable with other Western studies which have found that socio-demographic characteristics had no consistent relationship with compliance in general.[8,20]

Results of the present study found that the level of compliance increased with the improvement of the patient’s level of knowledge about diabetes. Another retrospective study done in Sharkia Governorate, Egypt, showed that good to adequately educated diabetics achieved better metabolic control than fair to poorly educated diabetics, as indicated by significantly lower HbA1c level in the former than the latter.[21] On the other hand, findings of a study conducted in China indicated that there was no association between the knowledge of diabetes and compliance.[22]

The results of the current study agree with those of other studies that show physician-patient communication to be frequently inadequate.[6,7,14]

The report of a study done in the USA in 2008 to evaluate adherence to oral diabetes medications in patients with Type 2 diabetes found that increasing age and the burden of comorbidity were associated with higher adherences to instruction on medication.[23] The present study demonstrated that a high percentage of patients who always complied with taking medication as prescribed (37.0%) and on time (37.7%) were 35 to <45 years old. These differences were statistically significant (*P*<0.05).

Results of the present study revealed a low percentage of patients (8%) who had poor glycemic control with HbAIc >8%. Results of a study conducted in two hospitals in Jeddah, KSA, showed that 42% of patients at King Abdul Aziz University Hospital and 46% of patients at Erfan Hospital had poor glycemic control.[23] This variation in the results of the studies may be because the current study was conducted on PHC patients who were regularly followed up and were given free medication while the second Saudi study was conducted on patients attending hospitals, but with complications and poor glycemic control.

Results of the present study revealed that patients who adhered to advice were more likely to achieve better glycemic control (high satisfactory score of HbA1c) than patients who were negligent. This agrees with the results of a recent study reported in 2008.[24]

## CONCLUSION AND RECOMMENDATIONS

Compliance of diabetic patients with most types of diabetes regimen was low. Increase in the level of patient’s knowledge of diabetes was associated with better compliance. The majority of patients who always took medications as prescribed, and on time and always adhered to dietary regimen had better glycemic control compared with others. None of the scores on doctor-patient relationship was satisfactory. There is a need for the improvement of doctor-patient relationship and an encouragement of patient’s compliance through the conduct of educational and training programs. The educational programs should be directed toward improving patient’s knowledge of diabetes in order to promote sound practice in the management of the disease. The training programs should be directed at health care providers in rural PHC units and aim at fostering better doctor-patient relationship. Further studies are needed to explore the effect of compliance with different aspects of the diabetic regimen on the quality of life of diabetic patients.
